# Polymeric Bioadhesive Patch Based on Ketoprofen-Hydrotalcite Hybrid for Local Treatments

**DOI:** 10.3390/pharmaceutics12080733

**Published:** 2020-08-04

**Authors:** Cinzia Pagano, Loredana Latterini, Alessandro Di Michele, Francesca Luzi, Debora Puglia, Maurizio Ricci, César Antonio Viseras Iborra, Luana Perioli

**Affiliations:** 1 Department of Pharmaceutical Sciences, University of Perugia, 06123 Perugia Italy; cinzia.pagano@unipg.it (C.P.); maurizio.ricci@unipg.it (M.R.); 2 Department of Chemistry, Biology and Biotechnology, University of Perugia, 06123 Perugia, Italy; loredana.latterini@unipg.it; 3 Department of Physics and Geology, University of Perugia, 06123 Perugia, Italy; alessandro.dimichele@collaboratori.unipg.it; 4 Civil and Environmental Engineering Department, University of Perugia, UdR INSTM, 05100 Terni, Italy; francesca.luzi@unipg.it (F.L.); debora.puglia@unipg.it (D.P.); 5 Department of Pharmacy and Pharmaceutical Technology, Faculty of Pharmacy, University of Granada, Campus of Cartuja, 18071 Granada, Spain; cviseras@ugr.es

**Keywords:** hydrotalcite, ketoprofen, hybrid, photostability, hydrogel film, bioadhesion

## Abstract

Ketoprofen (KET) represents one of the most common drugs used in the topical treatment of pain and inflammations. However, its potential is rather limited due to the very low solubility and photochemical instability. The local administration of KET by conventional products, such as gels, emulgels, creams, and foams, does not guarantee an efficacious and safe treatment because of its low absorption (due to low solubility) and its sensitivity to UV rays. The photodegradation of KET makes many photoproducts responsible for different adverse effects. In the present work, KET was intercalated into the lamellar anionic clay ZnAl-hydrotalcite (ZnAl-HTlc), obtaining the hybrid ZnAl-KET with improved stability to UV rays and water solubility in comparison to the crystalline form (not intercalated KET). The hybrid was then formulated in autoadhesive patches for local pain treatment. The patches were prepared by casting method starting from a hydrogel based on the biocompatible and bioadhesive polymer NaCMC (Sodium carboxymethycellulose) and glycerol as a plasticizing agent. The introduction of ZnAl-KET in the patch composition demonstrated the improvement in the mechanical properties of the formulation. Moreover, a sustained and complete KET release was obtained within 8 h. This allowed reducing the frequency of anti-inflammatory administration, compared to the conventional formulations.

## 1. Introduction

Ketoprofen (KET) is one of the most common NSAIDs (Nonsteroidal anti-inflammatory drugs) used for relieving pain in many acute and chronic conditions as musculoskeletal, tendinitis, strain, sprain, trauma, and arthritis [1,2]. However, its efficacy, especially in topical therapies, is impaired due to both the very poor water solubility (KET is labeled in class II of the biopharmaceutics classification system, BCS) and photochemical instability [3,4]. 

KET exposition to UV radiations, especially UVA, is responsible for phototoxic and photoallergic reactions [5,6]. These reactions are due to radical intermediates generated by KET-UV rays interactions responsible for DNA damage, mitochondria depolarization, and lysosomes destabilization [6,7,8]. Thus, KET topical administration by conventional formulations, such as gels, emulgels, creams, sprays, and foams, does not guarantee protection from UV radiation, exposing the patient to serious health problems [9]. Moreover, conventional formulations are responsible for a limited residence time of the drug in the application site. 

Thus, in order to exploit the benefits of KET and to overcome these problems, it is necessary to find a suitable formulation able to protect it from sunlight and to improve the residence time.

With this aim, many approaches have been purposed mainly based on KET entrapment in supramolecular structures as cyclodextrins, liposomes, niosomes, microparticles [10,11,12]. A further interesting approach is represented by the realization of host-guest complexes using an inorganic matrix as the anionic clay hydrotalcite (HTlc) [13]. This material shows the typical lamellar structure able to store anionic molecules (guest) in the nanosized interlamellar space (host) [14]. 

The general formula of synthetic HTlc is [M(II)_1−x_M(III)_x_(OH)_2_]^x+^(A^n−^_x/n_)^x−^ mS, where M(II) is a divalent metal cation (usually Mg, Zn); M(III) is a trivalent metal cation (usually Al, Fe); generally, the *x* value, M(III)/M(II) + M(III), ranges between 0.25 and 0.33; A^n−^ is an exchangeable inorganic or organic anion, which compensates the positive charge of the layer; m is the mol of solvent S, usually water, co-intercalated per mole of compound [13]. The intercalation of an organic molecule (drug) into HTlc lamellae allows obtaining a new inorganic-organic hybrid product with enhanced properties in terms of solubility [15,16], photoprotection [17,18,19], and physical and chemical stability [20]. These lamellar materials represent a valuable strategy in developing formulations with prolonged efficacy, thanks to the double control of the release and protection of the guest.

The aim of this work was to purpose a new formulation in which KET is stabilized from UV light, able to be applied on skin without adhesives, and to stay there for a prolonged time. Therefore, the photoprotective effect of the lamellar clay ZnAl-HTlc towards KET, once intercalated into the lamellae (ZnAl-KET), was investigated. Moreover, the performances of a self-adhesive/biocompatible patch loaded with the intercalation product ZnAl-KET were evaluated in terms of mechanical properties, bioadhesion, and drug release capability.

## 2. Materials and Methods

### 2.1. Materials

Ketoprofen acid form (KETH) and sodium salt (KET-Na) were purchased from Sigma-Aldrich (Milano, Italy). Glycerol and urea were purchased from Acef S.p.A. (Fiorenzuola D’Arda, Piacenza, Italy). Aluminum chloride esahydrate, calcium chloride, potassium dihydrogen phosphate, sodium hydrogen phosphate, polyvinilpyrrolidone, potassium carbonate (Carlo Erba, Cornaredo, Milano, Italy) were supplied by DueM (Perugia, Italy). Zinc oxide was supplied by Caelo (Hilden, North-Rhine Westphalia, Germany). Sodium carboxymethycellulose (NaCMC) was supplied by Hercules Inc.—Aqualon division (Wilmington, DE, USA). 

Ultrapure water was obtained by reverse osmosis process in a MilliQ system Millipore (Roma, Italy). Other reagents and solvents were of analytical grade and used without further purification.

### 2.2. Hydrotalcite Synthesis

ZnAl-HTlc in carbonate form was obtained by coprecipitation of Zn(II)-Al(III), accomplished by the hydrolysis of urea. The nitrate form ZnAl-HTlc-NO_3_ (ZnAl-NO_3_) was obtained, treating the solid with a diluted solution of the corresponding mineral acid [21].

### 2.3. Hybrid Preparation

The hybrid ZnAl-KET was prepared by ion-exchange mechanism according to previous work [15]. A carbon dioxide-free NaOH water solution 0.1 M was added to a suspension of KET (0.1 M). Then, ZnAl-NO_3_ was added to the obtained salt solution (molar ratio HTlc/KET 1:2). The suspension was kept under magnetic stirring (600 rpm) for 24 h at room temperature. The solid (ZnAl-KET) was recovered by centrifugation (A.L.C. centrifuge. mod. 4236A. Milano, Italy), washed with carbonate free water, and dried at room temperature under P_2_O_5_.

### 2.4. Hybrid Characterization

#### 2.4.1. X-ray Analysis

Powder X-ray diffraction patterns (XRPD) of powders were registered with a Philips X’Pert PRO MPD diffractometer (Malvern Panalytical, Royston, United Kingdom) operating at 40 kV and 40 mA, with a step size 0.03° 2theta and step scan 40 s, using Cu Kα radiation and an X’Celerator detector (Malvern Panalytical, Royston, United Kingdom).

#### 2.4.2. Metal Composition

Metal analyses were performed by Varian 700-ES series Inductively-Coupled Plasma-Optical Emission Spectrometers (ICP-OES, Varian Inc., Santa Clara, CA, USA) using solutions prepared by dissolving the samples in some drops of concentrated HNO_3_ solution and properly diluted.

#### 2.4.3. Thermogravimetric Analysis (TGA)

Thermogravimetric analysis (TGA) was performed by a Netzsch STA 449C apparatus (NETZSCH-Gerätebau GmbH, Selb, Germany) in air flow and heating rate of 10 °C/min to determine the weight loss (water and drug) as a function of increasing temperature.

#### 2.4.4. Differential Scanning Calorimetry (DSC) Analysis

DSC analyses were performed using an automatic thermal analyzer Mettler Toledo DSC821e (Mettler-Toledo S.p.A., Milano, Italy) and an indium standard for temperature calibrations. Holed aluminum pans were employed in the experiments for all samples, and an empty pan, prepared in the same way, was used as a reference. Samples of 3–6 mg were weighted directly into the aluminum pans, and the thermal analyses of samples were conducted, at a heating rate of 5 °C/min, from 25 to 200 °C.

#### 2.4.5. Scanning Electron Microscopy Analysis (SEM)

SEM micrographs were acquired by FE-SEM LEO 1525 ZEISS (Carl Zeiss Microscopy, Jena, Germany). The samples were prepared by deposition of the sample on conductive carbon adhesive tape and then metalized with chromium (8 nm) by sputtering. Elemental mapping was determined by energy dispersive X-ray analysis (EDX, Bruker Quantax, Billerica, MA, USA).

#### 2.4.6. Solubility Studies 

The solubility of KET from the crystalline acid form (KETH) and ZnAl-KET was measured in water, and the samples were prepared as follows: an excess of the sample (10 mg KETH and 24.10 mg of ZnAl-KET corresponding to 10 mg of KETH) was dispersed in 10 mL of ultrapure water under magnetic stirring (600 rpm) at 25 °C until equilibrium was achieved (48 h). The samples were filtered through a 0.45 μm nylon filter, suitably diluted, and analyzed by UV-VIS spectrophotometry. 

#### 2.4.7. Photochemical Analysis 

The photostability of the samples was spectrophotometrically investigated. Absorption spectra of the powder samples were recorded by a Varian Cary 4000 spectrophotometer (Varian, East Lyme, CT, USA), equipped with a 150-mm integration sphere, and a barium sulfate tablet was used as a reference. The recorded spectra were analyzed by the Kubelka-Munk equation. 

Steady-state irradiation experiments were performed using a Xenon lamp 150 W(Agilent, Santa Clara, CA, USA) as light source, the irradiation wavelength was selected by a monochromator, and a band-pass slit of 35 nm was used. The spectra analysis was carried out at different irradiation times.

### 2.5. Patches Preparation

The hybrid ZnAl-KET was formulated in patches by casting method [22] starting from the hydrogel composition optimized in previous work [23]:

ZnAl-KET: 1% *wt./wt*.

NaCMC: 2% *wt./wt*.

Glycerol: 10% *wt./wt*.

Water (CO_2_ free): 87% *wt./wt*.

The hybrid was mixed with NaCMC powder by mortar and pestle and then wetted with glycerol. The water, free from CO_2_ in order to avoid KET deintercalation, was added slowly until complete hydrogel formation. In order to remove the air incorporated during the mixing, the hydrogel was degassed in a conditioning planetary mixer (Thinky mixer ARE-250, Intertronics, Kidlington, England) at 2000 rpm for 10 min. The patches were prepared by casting 3.5 g of the hydrogel in circular silicon molds (diameter 3 cm) and then placed in a ventilated oven at 37 °C for 24 h.

### 2.6. Patches Characterization

#### 2.6.1. Weight and Thickness Measurement

Patches (circles, area: 7.065 cm^2^) were weighted by a Mettler-Toledo weighing balance, model XS205 (Milano, Italy). The thickness was measured in three different points of the patch by an electronic micrometer (QuantuMike IP 65 Coolant Proof, Mitutoyo, Takatsu-ku, Japan).

#### 2.6.2. Mechanical Characterization 

The tensile tests were performed by using a digital microprocessor instrument Llyod LR30K (Lloyd instruments, Bognor Regis, UK). The patches were cut in portions 100 × 10 mm (UNI ISO 527) to have a useful length of 50 mm. The experiment was performed at 5 mm/min, cell load 50 N. The two ends of the patch were fixed with clamps to the dynamometer. The sample was subjected to tensile stress until deformation and break. Maximum stress (*σ_max_*), deformation at the break for maximum stress (*ε _at σmax_*), and elastic modulus (*E_Young_*) values were calculated from the stress-strain curves. Before the test, the patches were equilibrated for 1 week under silica gel (relative humidity, RH 30%). The results were an average of five measurements (n = 5). 

#### 2.6.3. Ex Vivo Adhesion Studies

Patch adhesion force was assessed using pig skin samples (from shoulder region), obtained from large white pigs weighing ∼165–175 kg, furnished by Veterinary Service of ASL N.1 Città di Castello (Perugia, Italy), and used within 12 h from pig death [22]. The ex vivo adhesion force was measured by a dynamometer (Didatronic, Treni, Italy). The patch (2 × 2 cm) was attached to support, connected to the dynamometer, using cyanoacrylate glue. A piece of porcine skin tissue (4 × 4 cm) was fixed with cyanoacrylate glue on the surface of glass support placed in a thermostatic bath at 32 °C ± 0.5. The free side of the patch was wetted with 100 μL of phosphate buffer pH 5.50 and put in contact with the skin sample by applying a light force for 20 s. The force necessary for patch detachment to skin was measured and expressed as the average of three measurements (n = 3).

#### 2.6.4. In Vitro Release 

In vitro KET release, from ZnAl-KET and ZnAl-KET dispersed in the patch, was evaluated by dissolution testing for transdermal patches according to the European Pharmacopoeia (Ph. Eur. 10th Ed.). Precisely, the apparatus II of the dissolution test was used, positioning an extraction cell (Ph. Eur. 10th Ed.) at the bottom of the vessel. 

The central part of the cell formed a cavity (depth of 2.6 mm, diameter 27.0 mm) to hold the patch. On the top of the cell was positioned a cover with a central opening of 20.0 mm obtaining a release surface of 3.14 cm^2^. The cell was positioned at the bottom of the vessel with the cover facing upwards and at a distance of 25.0 ± 2.0 mm from the paddle blade. The test was carried out for 24 h by working at 40 rpm at 32.0 ± 0.5 °C in sink conditions and using phosphate buffer pH 5.50 (Ph. Eur. 10th Ed.) as a dissolution medium (400 mL). At predetermined intervals, 2 mL of sample was withdrawn from the vessel and replaced by the same amount of fresh dissolution medium. 

#### 2.6.5. Quantitative Analysis

KET quantification was performed using a UV-VIS spectrophotometer (8453 Agilent, Santa Clara, CA, USA). The quantification was made using a calibration curve in phosphate buffer pH 5.50 previously prepared (λ_max_ = 261 nm, r = 0.99), using phosphate buffer pH 5.50 as blank. Experiments were performed in triplicate, and the error expressed as standard deviation (±SD).

#### 2.6.6. Statistical Analysis

Results were reported as mean ± standard deviation (mean ± SD). A one-way ANOVA test was used for statistical analysis. Differences were considered statistically significant for *p* < 0.05.

## 3. Results

### 3.1. Hybrid Preparation and Characterization

**Scheme 1:**
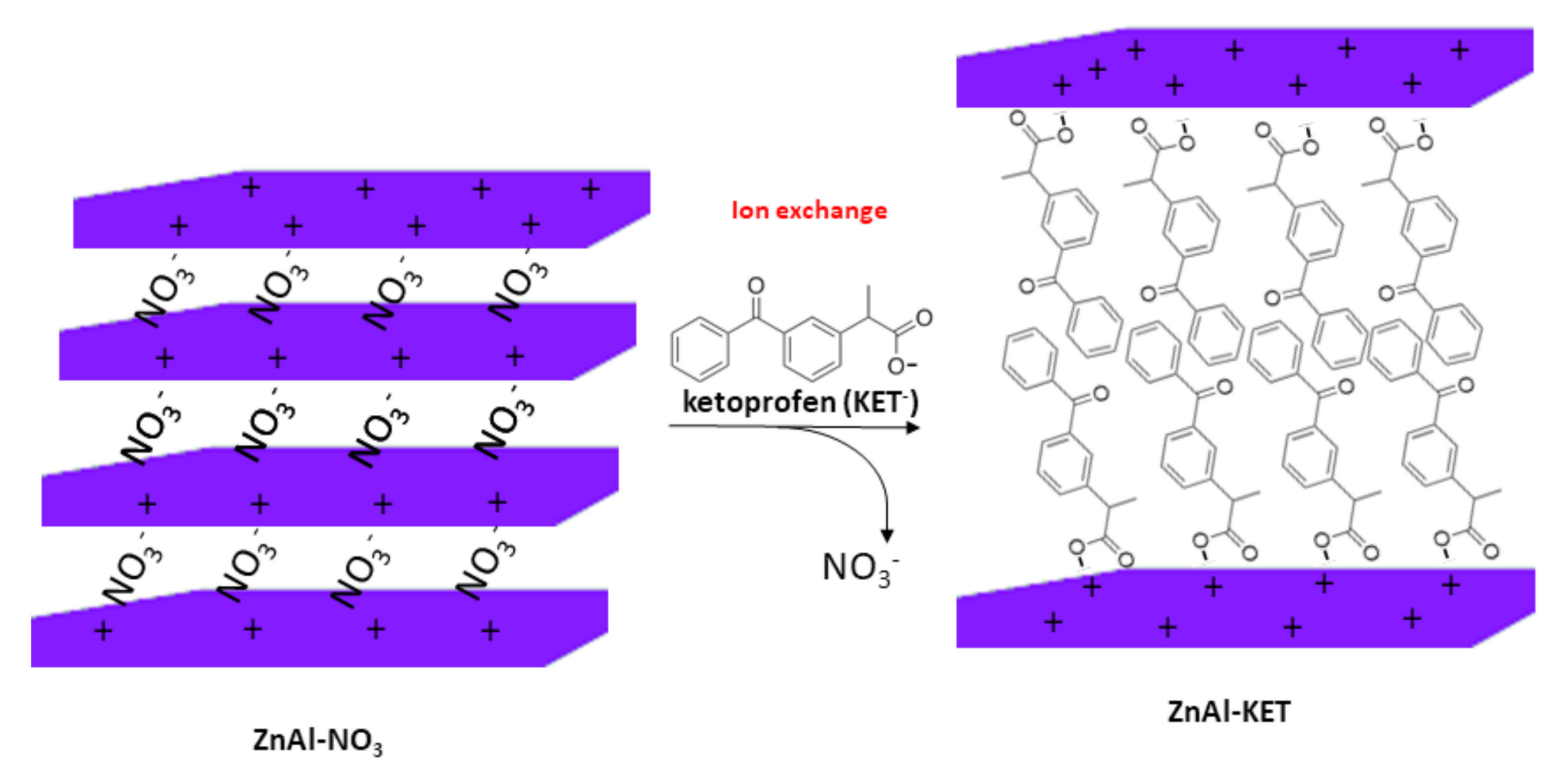
Schematic representation of the ion exchange between NO_3_^−^ ions in the pristine ZnAl-HTlc (ZnAl-hydrotalcite) and KET^−^ (ketoprofen).

The hybrid ZnAl-KET was obtained by ion exchange mechanism (Scheme 1) using ZnAl-NO_3_, having the following formula obtained by ICP-OES analysis: [Zn_0.70_ Al_0.30_ (OH)_2_](NO_3_)_0.30_·0.4 H_2_O.

**Figure 1:**
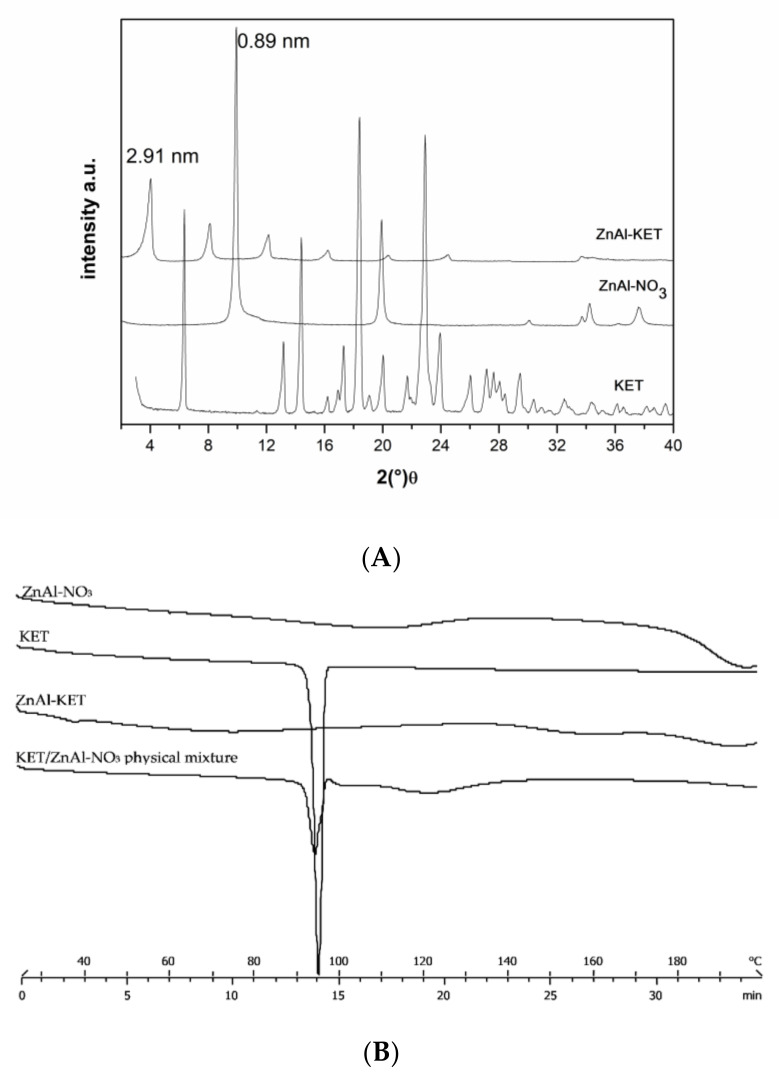
(**A**) XRPD pattern (X-ray diffraction patterns) of crystalline ketoprofen (KET) (acid form), pristine ZnAl-NO_3_, and the hybrid ZnAl-KET. (**B**) Thermal profiles measured by differential scanning calorimetry of ZnAl-NO_3_, crystalline KET, ZnAl-KET, and KET/ZnAl-NO_3_ physical mixture.

The XRPD analysis confirmed KET^-^ intercalation into HTlc lamellae. In fact, the hybrid pattern showed a reflection at 2.19 nm, which was increased in comparison to pristine ZnAl-NO_3_ that shows a reflection at 0.89 nm, typical of nitrate anion (Figure 1A). Combining the results coming from ICP-OES and TGA analyses, the final formula was calculated as [Zn_0.70_ Al_0.30_ (OH)_2_] (KET)_0.30_·0.98 H_2_O (ZnAl-KET); drug loading was 41.89% *wt./wt*. 

KET intercalated into HTlc in the anionic form (KET^−^) showed a different solid-state compared to the pristine crystalline form. In fact, the reflection of the crystalline form disappeared in the intercalation product XRPD spectrum, testifying the lack of the crystalline form (Figure 1A). This was confirmed also by the thermal profile obtained by differential scanning calorimetry analysis. In fact, while crystalline KETH (alone) showed a sharp endothermic peak at 94 °C, corresponding to the melting point, this was not detectable in the ZnAl-KET profile, meaning that the crystallinity form was not present in this product (Figure 1B).

**Figure 2:**
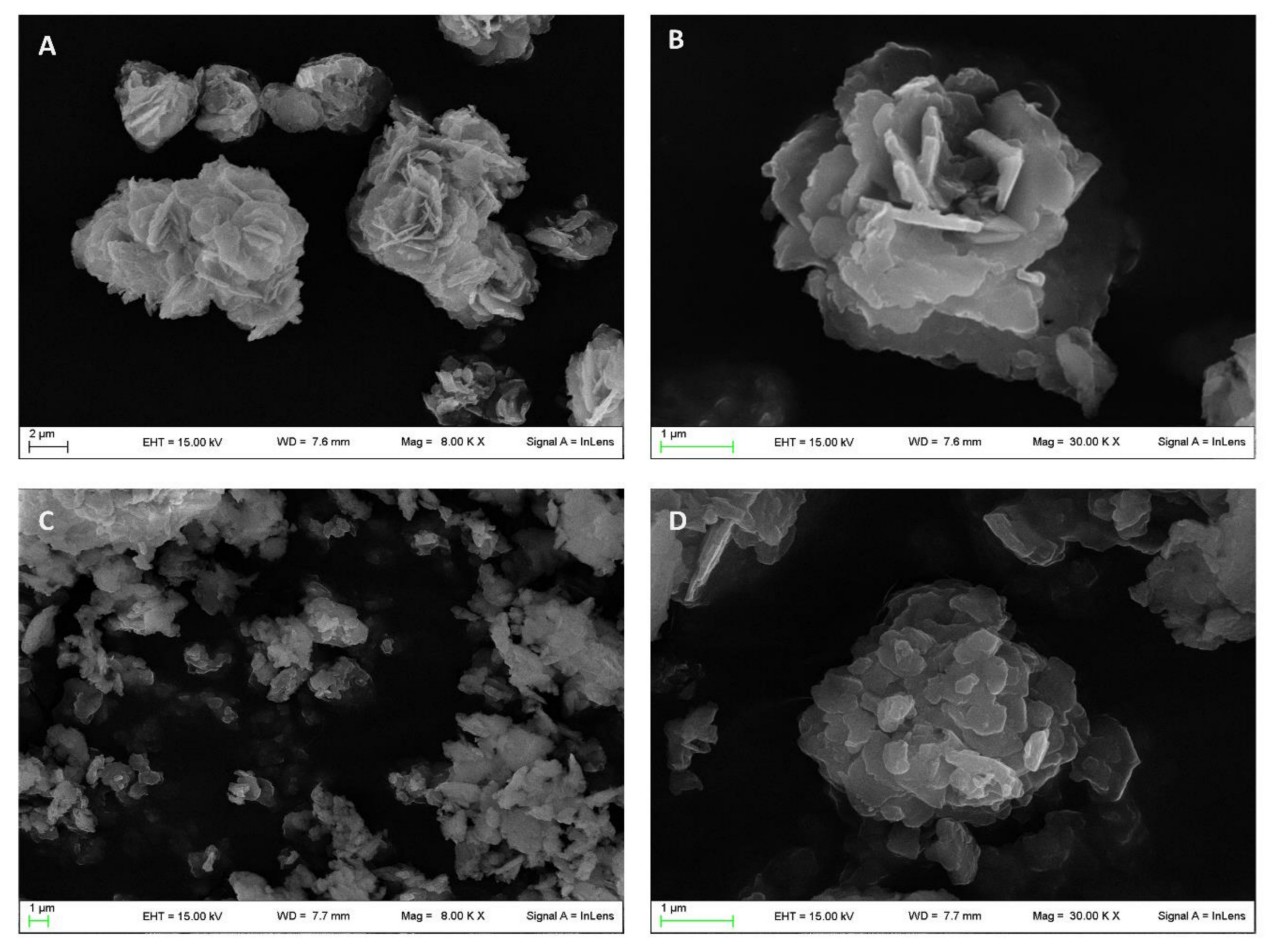
Micrographs obtained by scanning electron microscopy of pristine ZnAl-NO_3_ (**A**,**B**) and ZnAl-KET (**C**,**D**).

The morphological analysis carried out by scanning electron microscopy (SEM) showed the typical desert-like rose structure of pristine ZnAl-NO_3_ crystals (Figure 2A,B) [24] having dimensions in the range of 1–10 µm. The intercalation procedure induced the modification of this morphology, as can be observed from the micrographs reported in Figure 2C,D appearing fragmented with rounded edges, while the dimensions were maintained in the same range of the raw ZnAl-NO_3_.

### 3.2. Apparent Solubility Studies

The solubilization rate of KET from the crystalline form (acid form) from the hybrid (ZnAl-KET) was evaluated in vitro method in order to evaluate if the intercalation procedure allows enhancing this property. The obtained results showed that crystalline KET (acid form) had a limited solubilization rate, as testified by the concentration value measured (12 ± 0.15 µg/mL). This result was in accordance with the literature data [25]. On the other hand, the results obtained from ZnAl-KET were very different; in fact, a concentration of 106.00 ± 0.20 µg/mL was measured. This result can be explained by taking into account that KET is intercalated into HTlc lamellae as an anion in the molecular form. Thus, when ZnAl-KET makes contact with the water, KET^-^ anions are exchanged and replaced by OH^−^ (deriving from water molecules dissociation) in the interlayer space. Once in the medium, KET^-^ anions are protonated obtaining a free dissolved molecules. 

### 3.3. Photochemical Characterization 

**Figure 3:**
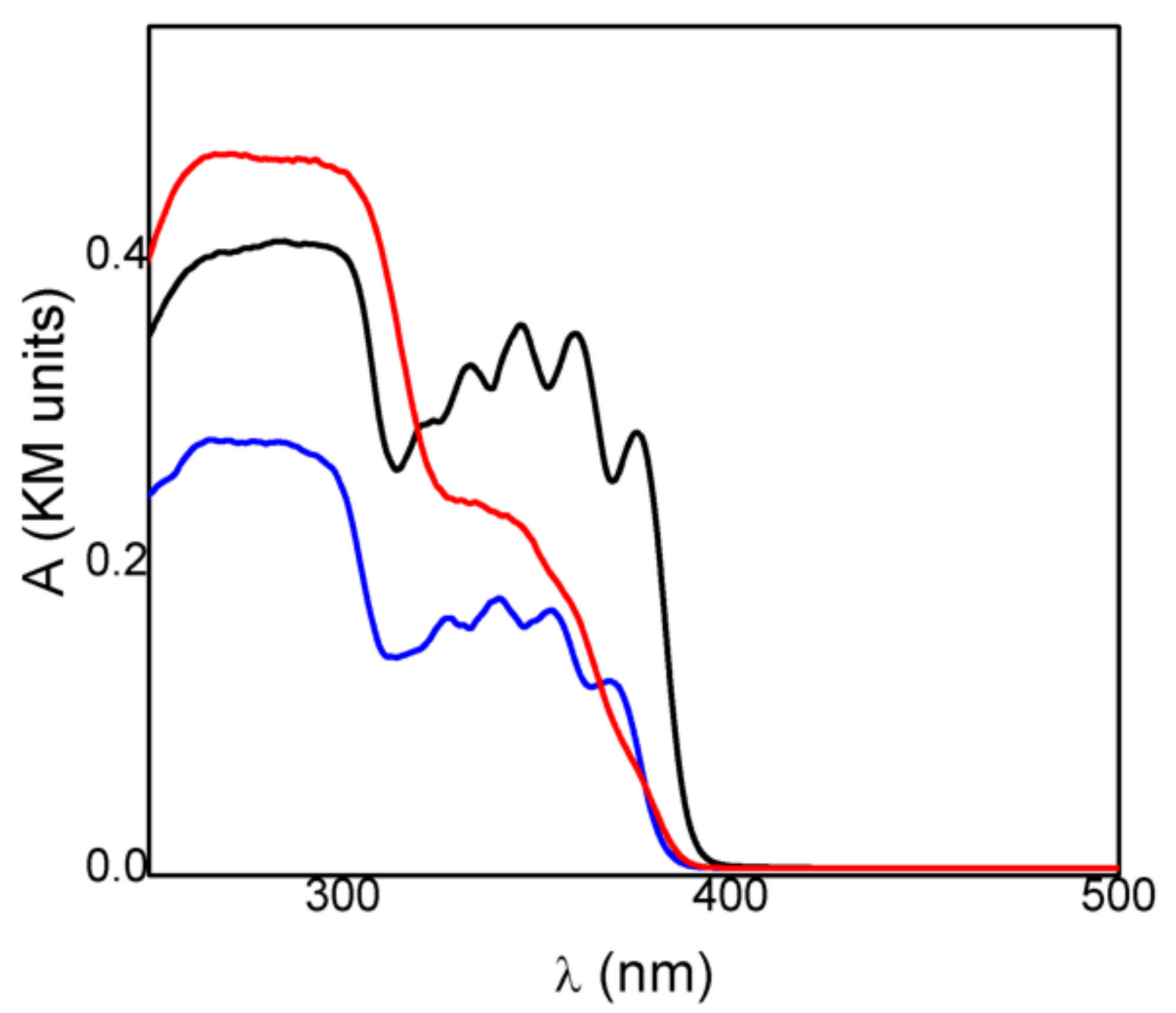
Absorption spectra of crystalline KET in acid form (KETH) (black line), KET-Na (blue line), and ZnAl-KET (red line).

Spectrophotometric measurements gave insight into the radiation frequencies absorbed by the drug and its intercalated compound and could be used to achieve information on the photostability of the materials. The absorption spectrum of crystalline KET in acid form (KETH) presented a maximum at 280 nm and a broad and structured band in the 320–390 nm (Figure 3). Deprotonation of the drug did not change its absorption spectrum; in fact, the KET-Na (Figure 3) showed a spectrum very similar to that obtained for KETH, probably because the acid functionality is not conjugated with the chromophoric moiety. On the other hand, the spectrum of the intercalated sample ZnAl-KET showed the main band centered at 280 nm and a shoulder without any structures at about 330 nm (Figure 3). The different spectra recorded for KET-Na and ZnAl-KET indicated that the interactions with the inorganic matrices and/or the interactions between chromophores modified the electronic distribution in the drug.

**Figure 4:**
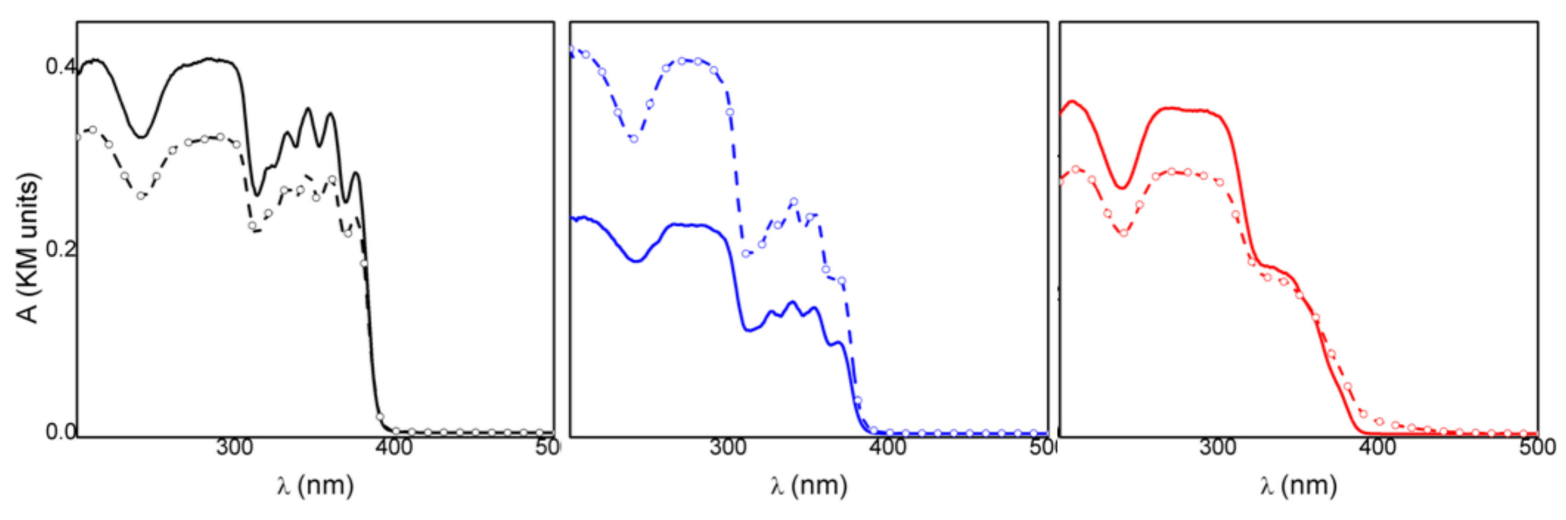
Absorption spectra of KETH (black line), KET-Na (blue line), and ZnAl-HTlc-KET (red line) before and after 120′ irradiation at 330 nm.

The spectrophotometric analysis conducted on the samples under investigation before and after irradiation at 330 nm enabled us to evaluate and compare their photostability. In all cases, after 2 h of irradiation, the optical density changed (ΔOD) in the 250–400 nm range and was detected even if the relative variations were different (Figure 4). In particular, KET showed a smaller variation, compared to the other samples, in the whole spectral range investigated (black line Figure 4, Table 1). This behavior indicated that KETH had a higher photostability, in agreement with the literature data [26], where the photodecomposition of ketoprofen is reported to be pH-dependent, and a higher photodegradation is observed when the drug is in the anionic form. Therefore, KETH was not a good reference for the intercalated samples. KET-Na showed a remarkable increase in the absorption upon corresponding irradiation (blue line Figure 4, Table 1); this indicated that the deprotonated drug had a photostability lower than KETH, also when in powder form. Since the drug was intercalated in an anionic matrix, KET-Na was considered a good reference for assessing the photochemical behavior of the intercalation compound.

When ZnAl-KET was exposed to the 330 nm radiation (red line Figure 4) in the same experimental conditions, a decrease in the absorption at wavelengths below 360 nm was observed, while at longer wavelengths, an increase in optical density was detected. This behavior suggested that the drug underwent different photochemical processes in the inorganic matrix. However, the optical density changes of the intercalation compound were small compared to those observed for KET-Na, as shown by the data reported in Table 1.

The comparison of the data suggested that the hybrid ZnAl-HTlc was able to photoprotect the deprotonated form of KET. The photoprotection mechanism can be due to a physical filter effect and to a modification of the photochemical reaction paths. Indeed, it has been reported that KET carboxylate excitation produces mainly the triplet state, which undergoes a rapid and efficient photodecarboxylation reaction [27]. The intercalation in the layered solid could reduce the efficiency of the decarboxylation step due to space confinement of the species.

### 3.4. Patch Characterization

Previous work highlighted that patches realized starting from a hydrogel based on NaCMC (2% *wt./wt.*) as bioadhesive polymer, glycerol (10% *wt./wt.*) as a plasticizing agent, hydrotalcite ZnAl-NO_3_ (1% *wt./wt.*), and water (until 100%) shows suitable mechanical properties for topical use [23]. Using this composition, a new hydrogel was prepared using ZnAl-KET 1% *wt./wt.* in place of ZnAl-NO_3_. The patch was prepared to cast the hydrogel in a circular mold, obtaining a final formulation with a surface area of 10.17 cm^2^, a final weight of 0.54 g ± 0.01 (0.0532 g/cm^2^), and a thickness of 414 µm ± 7.42. Considering the loading percentage of ZnAl-KET (41.89% *wt./wt.*), the amount of KET/surface was measured to be 1.44 mg/cm^2^. 

**Figure 5:**
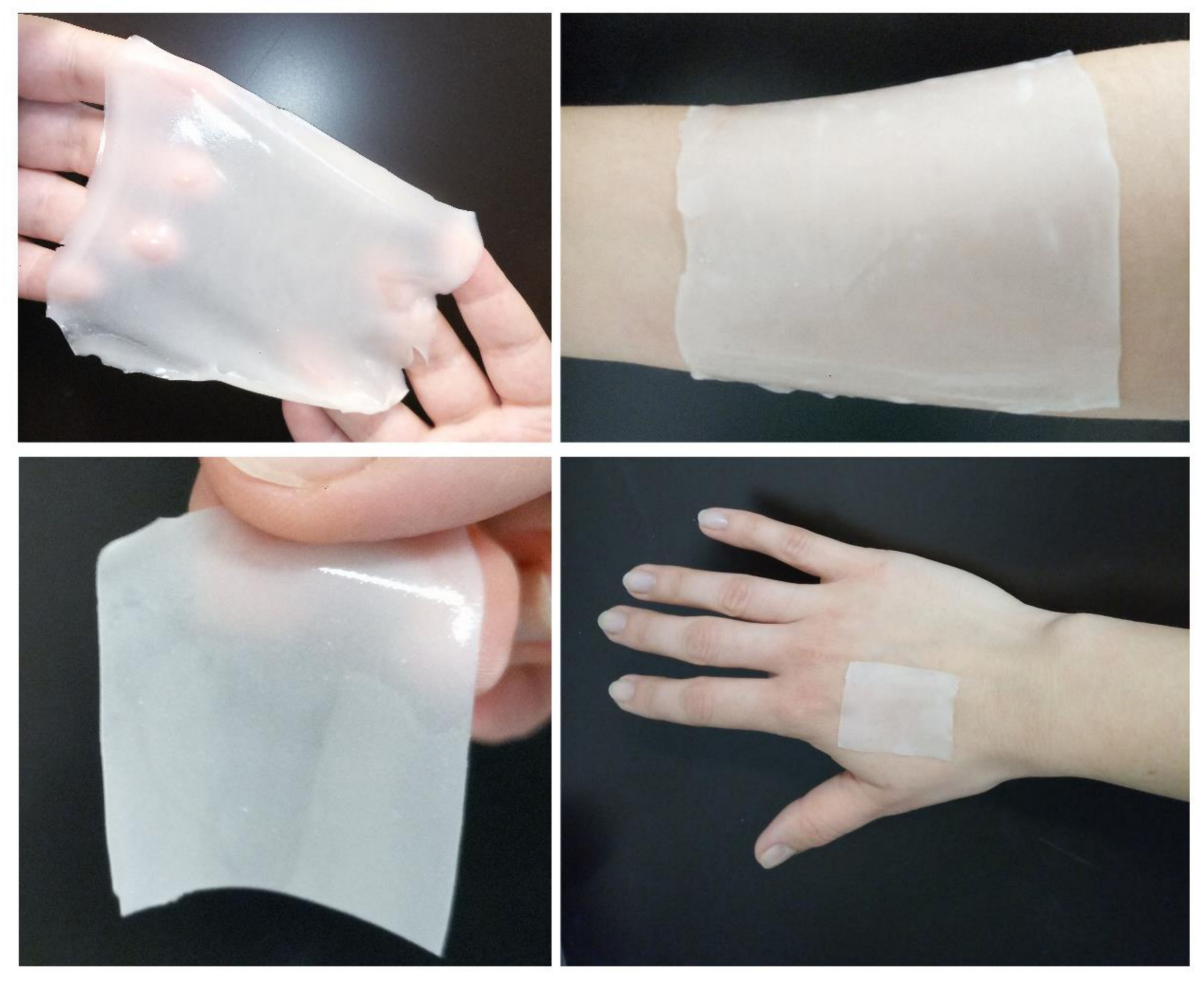
Examples of different sizes and shapes on which it can be applied the patch loaded with ZnAl-KET.

Taking into account this datum, the patch can be prepared in different sizes to be applied on surfaces of different dimensions, as demonstrated in the pictures reported in Figure 5.

#### 3.4.1. Morphological Characterization

**Figure 6:**
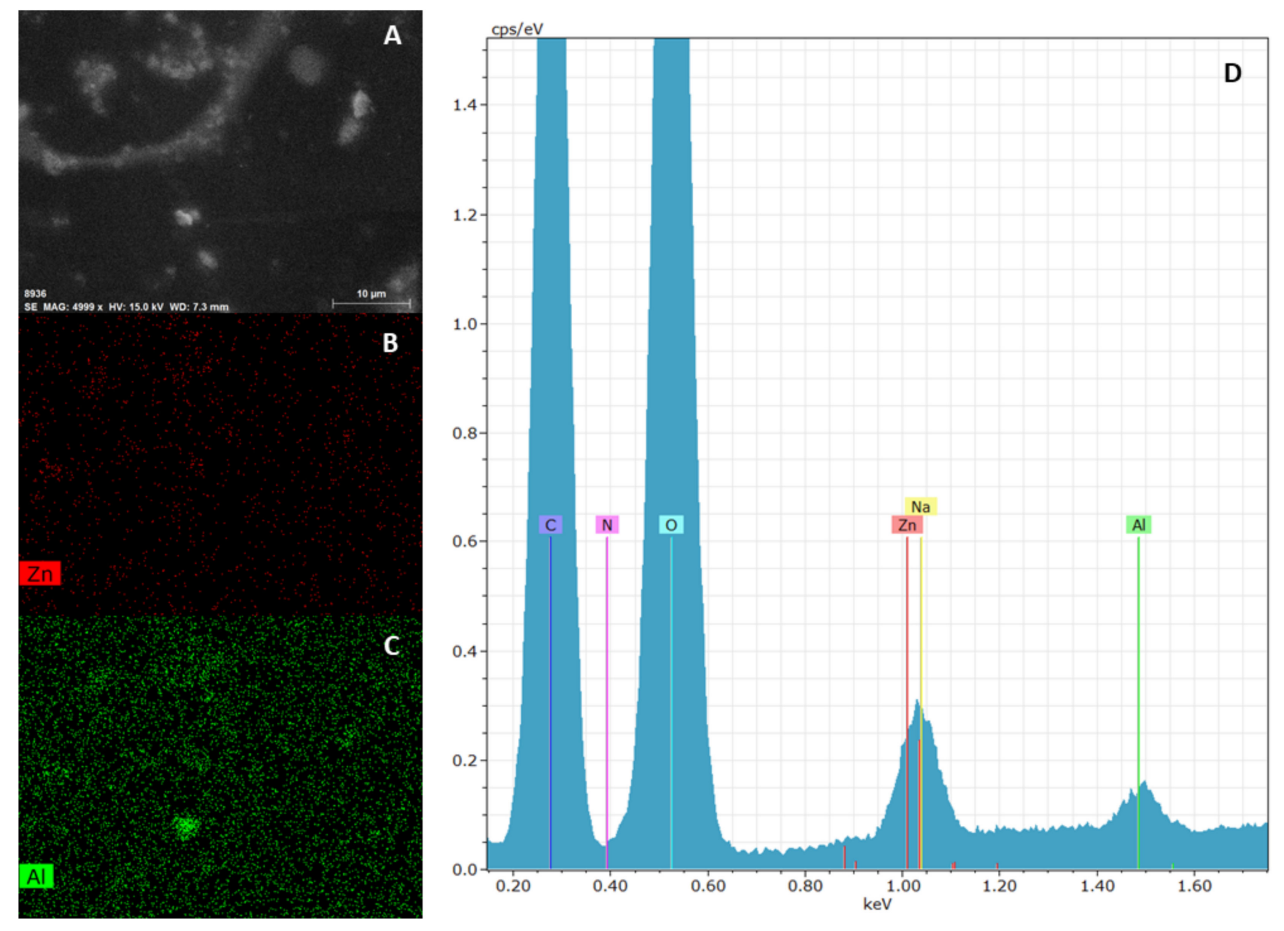
SEM analysis of ZnAl-KET loaded patch: micrograph (**A**), zinc mapping (**B**), aluminum mapping (**C**) and edx spectrum (**D**). Magnification 4999 X.

The elemental mapping on ZnAl-KET loaded patch obtained by EDX analysis showed that the hybrid was homogeneously dispersed in the polymeric matrix (Figure 6). This result suggested that the casting method used for patch preparation allowed to obtain a homogeneous final product, and this was very important as a guarantee that the drug (KET as a hybrid) was distributed homogeneously on the whole surface. Moreover, the patch showed a wrinkled surface due to the presence of ZnAl-KET crystals.

#### 3.4.2. Mechanical Characterization

**Figure 7:**
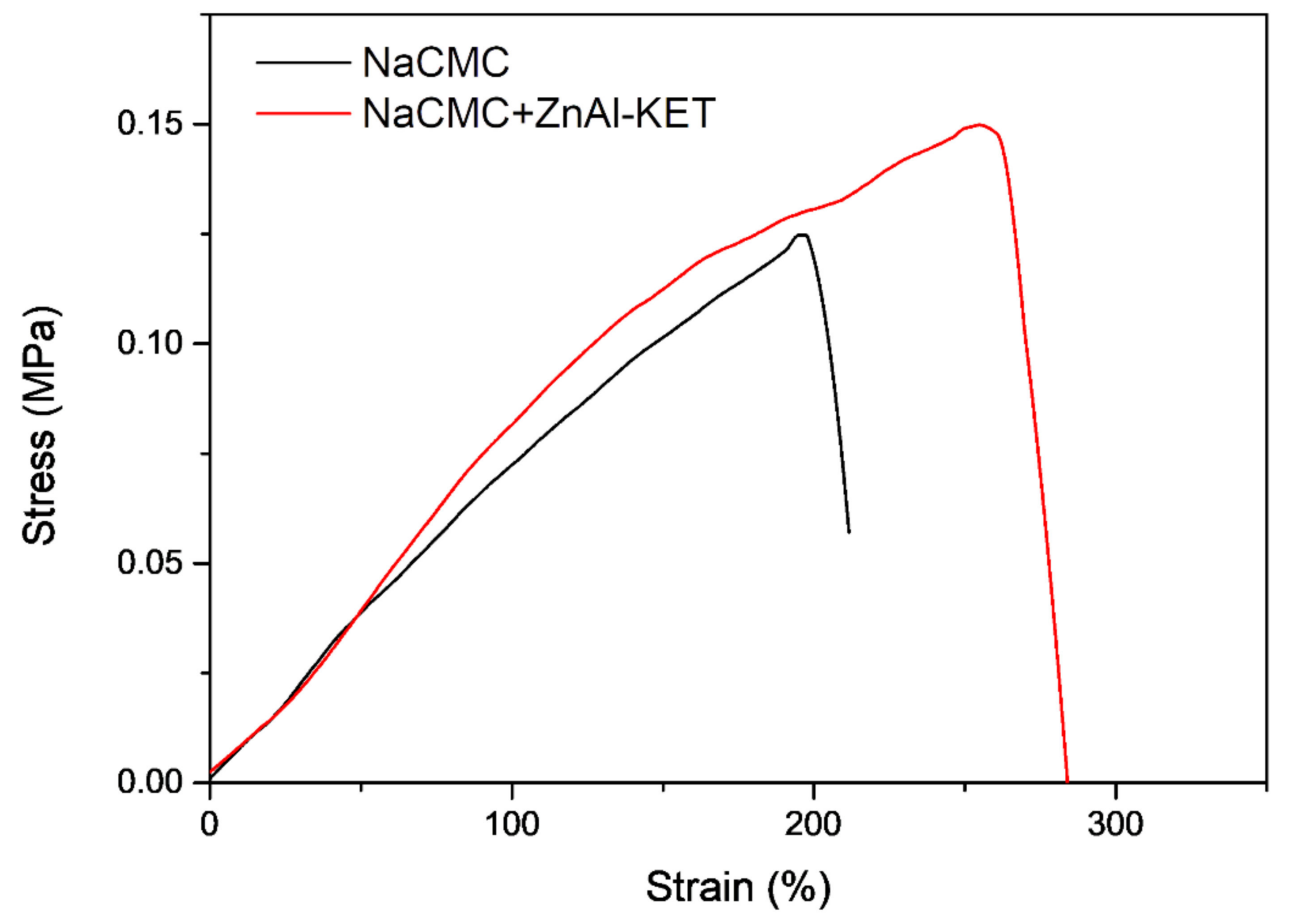
Stress–strain curves of NaCMC patch (NaCMC) and patch loaded with ZnAl-KET (NaCMC + ZnAl-KET).

The presence and effect of ZnAl-KET in the NaCMC matrix were investigated in terms of mechanical properties. Mechanical parameters of the two patches, NaCMC (blank) and NaCMC loaded with ZnAl-KET, were estimated from stress-strain curves obtained by performing tensile tests at RT (Figure 7). Data are summarized in Table 2, while the stress-strain curves are reported in Figure 6. As already reported in Perioli et al. [23], the introduction of lamellar ZnAl-NO_3_ at 1% *wt.* in NaCMC, even in a small amount, was able to increase the mechanical properties of reference matrix. Specifically, at low humidity conditions, the produced patch showed increased tensile strength but limited deformability at the break, as already observed by Yadollahi et al. [28]. The ductility of the NaCMC film was indeed here recovered and greatly improved when KET was intercalated in ZnAl-NO_3_. The introduction of the drug into ZnAl-HTlc might enhance interfacial adhesion and compatibility between the NaCMC matrix and the layered nano clay, thus increasing tensile strength [29] and positively affecting the ductility of the produced patch. In particular, it could be considered that the presence of KET inhibited the possible restacking of the nano sheets that could appear in unmodified HTlcs, by maintaining the exfoliated state in water-soluble NaCMC before casting [30].

### 3.5. Ex Vivo Adhesion Studies

Patch loaded with ZnAl-KET showed a bioadhesion force of 0.513 ± 0.015 N vs. 0.411 ± 0.011 N measured for the same patch without HTlc (blank). This increase was probably due to the wrinkled surface of the loaded patch, able to promote the contact and adhesion with the skin surface. Moreover, it can be hypothesized that ZnAl-KET establishes hydrophilic interactions (hydrogen bonds) with NaCMC chains involving −OH and C=O groups. This should improve the hydrophobicity of patch surface due to the exposition of NaCMC lipophilic groups (as-CH_3_) freely available to bind the skin. In fact, the binding to the stratum corneum is mainly attributable to hydrophobic interactions. 

### 3.6. Release Studies

**Figure 8:**
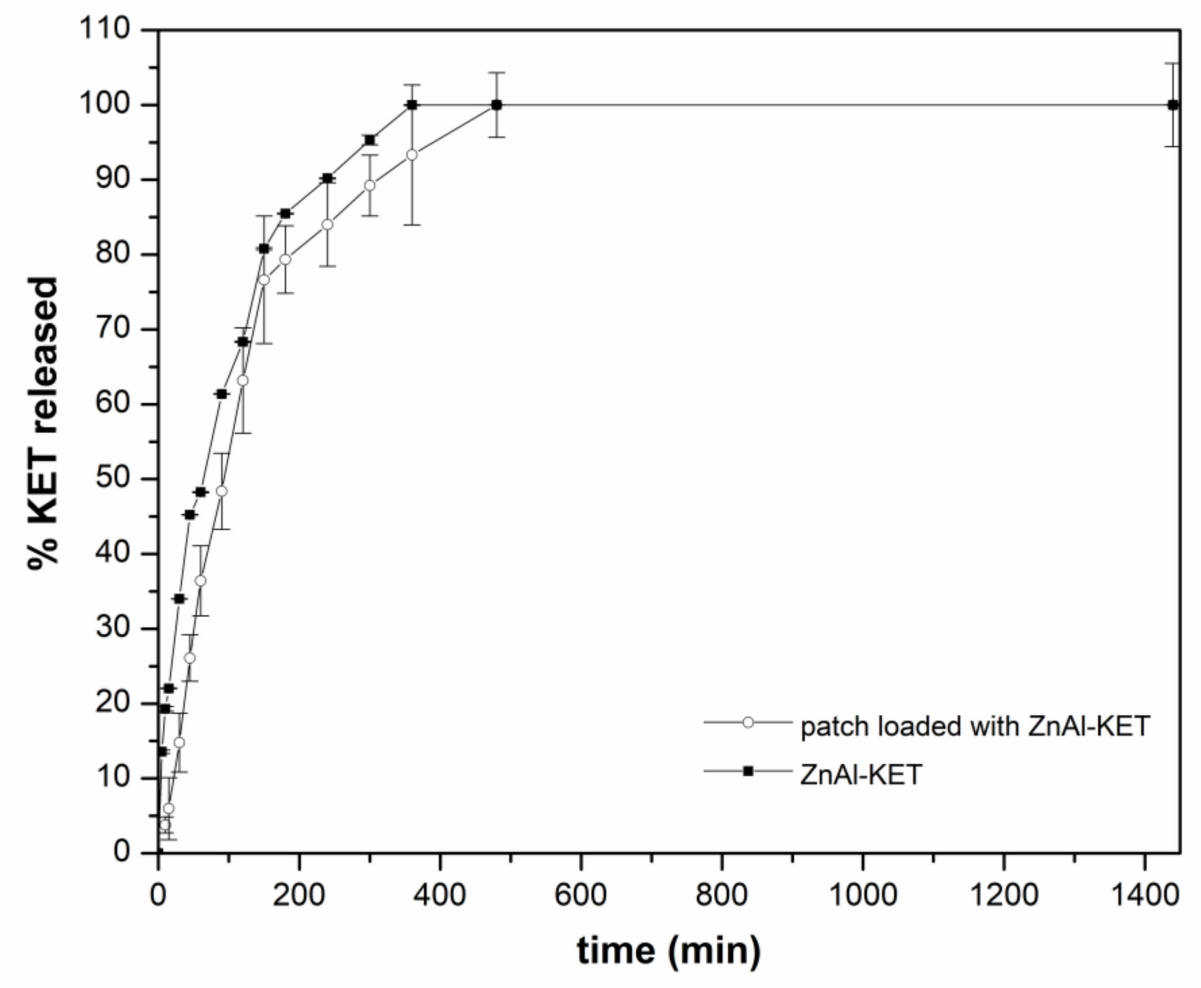
The release profile of KET from ZnAl-KET and from the patch loaded with ZnAl-KET (*p* < 0.05).

The release capacity of the patch loaded with ZnAl-KET was evaluated using the in vitro method for transdermal patches, according to Ph. Eur. 10th Ed. As shown in Figure 8, a sustained release of KET was obtained from the formulation reaching 6% after 5 min, 15% after 30 min, and ~36% after 60 min. The complete release was obtained within 480 min; from this point, a steady state was observed. The same assay performed on ZnAl-KET (not formulated in the patch) showed that the amount of KET released in the first 300 min from the hybrid was higher (after 10 min 13.5% vs. ~4%; after 45 min 45% vs. 26%; after 300 min 93% vs. 89%) compared to the patch. This suggested the effect of the polymeric matrix (in the case of the patch) in controlling the drug diffusion. 

The kinetic of KET release from the patch can be explained considering two main mechanisms, (i) the ion exchange between KET^-^ stored in the HTlc interlamellar space and phosphate anions present in the dissolution medium and (ii) the effect of NaCMC polymeric network, able to modulate KET diffusion in the bulk solution.

The kinetic of KET release was deeply investigated by processing the in vitro release by the following mathematical models: zero-order, first-order, and Higuchi [31,32]. When the zero-order model describes the release rate, the latter is not dependent on the concentration; the first-order model describes the release rate concentration-dependent, and the Higuchi model explains the release based on Fickian diffusion (time-dependent). Moreover, as KET was homogeneously dispersed in the NaCMC network of the patch as intercalation product (ZnAl-KET), the influence of HTlc on the release was evaluated by the kinetic model (ion exchange resins) proposed by Bhaskar et al. [33], applied to exchangeable matrices.

The obtained results from the patch (Table 3) showed that the best fitting was obtained both for Higuchi (R^2^ = 0.98) and Bhaskar models (R^2^ = 0.98). This suggested that these two models were the most suitable to describe KET release from the patch. The amount of KET molecules available to cross the NaCMC polymeric network by time-dependent diffusional mechanism (Higuchi kinetic) was controlled by the ion exchange mechanism between the intercalated KET^-^ and phosphate ions of the dissolution medium. The good fitting obtained in the case of the Bhaskar model suggested that this process had a remarkable role in conditioning the diffusion rate. 

ZnAl-KET (Table 3) showed a good fitting for the Bhaskar model (R^2^ = 0.97), confirming that, in this case, the ion exchange between KET^-^ and phosphate ions was the mechanism driving the dissolution rate. 

## 4. Conclusions

KET was intercalated into the lamellar anionic clay ZnAl-NO_3_ to obtain the hybrid ZnAl-KET and then formulated in an authoadhesive patch as an alternative to conventional products (gels, foams, creams) for local treatments. 

The performed studies highlighted that the intercalation is a valuable technology and advantageous to improve KET water solubilization rate and stability to UV radiations.

The hybrid ZnAl-KET was loaded in a bioadhesive polymeric patch, prepared using NaCMC, as an alternative to conventional products for local pain treatment. 

It was demonstrated that the presence of ZnAl-KET crystals, homogeneously dispersed in the polymeric matrix, was able to improve the mechanical patch properties. Moreover, a sustained release was observed by in vitro method, suggesting that the planned formulation could assure prolonged KET release. The proposed patch was bioadhesive, allowing both a high residence time in the application site and easy removal by washing, avoiding the discomfort of adhesives used in conventional patches, responsible for pain during the removal.

The patch formulation is also versatile and practical to use. It can be prepared or cut, if needed, in various sizes and shapes, becoming useful to be used both for large and small surfaces.

Moreover, the production of such a formulation is easily scalable. 

## Figures and Tables

**Table 1:** ΔOD measured at different λ_obs_ upon 2 h irradiation of the samples at 330 nm.

Sample	ΔOD [λ_obs_ = 280 nm]	ΔOD [λ_obs_ = 340 nm]
KETH	2%	3%
KET-Na	44%	45%
ZnAl-KET	24%	5%

ΔOD: optical density changes; λ_obs_: observation wavelengths

**Table 2:** Mechanical properties of NaCMC patch and patch loaded with ZnAl-KET.

Formulations	*σ_max_* (MPa)	*ε_at σmax_* (%)	*E_Young_* (MPa)
NaCMC	0.134 ± 0.014	187 ± 18	0.068 ± 0.008
NaCMC + ZnAl-KET	0.149 ± 0.013	252 ± 17	0.068 ± 0.009

NaCMC: NaCMC patch; NaCMC + ZnAl-KET: patch loaded with ZnAl-KET; *σ_max_*: maximum stress; *ε _at σmax_*: deformation at the break for maximum stress; *E_Young_*: elastic modulus.

**Table 3:** Equations and *R^2^* values obtained by the application of the kinetic mathematical models (zero-order, first-order) and ion exchange resins for loaded patch and ion exchange resins for ZnAl-KET.

Sample	M_t_/M_∞_ = kt	M_t_/M_∞_ = kt^0.5^	M_t_/M_∞_ = 1 − e^−kt^	M_t_/M_∞_ = 1 − e^−k 0.65^
	Zero-order kinetic	Higuchi kinetic (release 0–60%)	First order kinetic	Ion exchange resins
ZnAl-KET	-	-	-	y = −0.0322x + 0.1089 R^2^ = 0.97
patch	y = 0.3292x + 9.8281 R^2^ = 0.885	y = 6.8906x − 18.739 R^2^ = 0.98	y = 0.3292x + 9.8281 R^2^ = 0.88	y = −0.027x + 0.1425 R^2^ = 0.98

M_∞:_ amount of drug at the equilibrium state; M_t_: amount of drug released over time t, k: release velocity constant; n: exponent of release (related to the drug release mechanism) in function of time t; e: Euler’s number.
